# Identifying Interventions to Improve Diagnostic Safety in Emergency Departments: Protocol for a Participatory Design Study

**DOI:** 10.2196/55357

**Published:** 2024-06-21

**Authors:** Woosuk Seo, Sun Young Park, Zhan Zhang, Hardeep Singh, Kalyan Pasupathy, Prashant Mahajan

**Affiliations:** 1 School of Information University of Michigan Ann Arbor, MI United States; 2 School of Information, Stamps School of Art and Design University of Michigan Ann Arbor, MI United States; 3 Seidenberg School of Computer Science and Information Systems Pace University New York, NY United States; 4 Center for Innovations in Quality, Effectiveness and Safety (IQuESt), Michael E. DeBakey Veterans Affairs Medical Center and Baylor College of Medicine Houston, TX United States; 5 Biomedical and Health Information Sciences University of Illinois Chicago Chicago, IL United States; 6 Department of Emergency Medicine University of Michigan Medical School Ann Arbor, MI United States

**Keywords:** emergency departments, participatory design, diagnostic process, multilevel interventions, sociotechnical interventions, mobile phone

## Abstract

**Background:**

Emergency departments (EDs) are complex and fast-paced clinical settings where a diagnosis is made in a time-, information-, and resource-constrained context. Thus, it is predisposed to suboptimal diagnostic outcomes, leading to errors and subsequent patient harm. Arriving at a timely and accurate diagnosis is an activity that occurs after an effective collaboration between the patient or caregiver and the clinical team within the ED. Interventions such as novel sociotechnical solutions are needed to mitigate errors and risks.

**Objective:**

This study aims to identify challenges that frontline ED health care providers and patients face in the ED diagnostic process and involve them in co-designing technological interventions to enhance diagnostic excellence.

**Methods:**

We will conduct separate sessions with ED health care providers and patients, respectively, to assess various design ideas and use a participatory design (PD) approach for technological interventions to improve ED diagnostic safety. In the sessions, various intervention ideas will be presented to participants through storyboards. Based on a preliminary interview study with ED patients and health care providers, we created intervention storyboards that illustrate different care contexts in which ED health care providers or patients experience challenges and show how each intervention would address the specific challenge. By facilitating participant group discussion, we will reveal the overlap between the needs of the design research team observed during fieldwork and the needs perceived by target users (ie, participants) in their own experience to gain their perspectives and assessment on each idea. After the group discussions, participants will rank the ideas and co-design to improve our interventions. Data sources will include audio and video recordings, design sketches, and ratings of intervention design ideas from PD sessions. The University of Michigan Institutional Review Board approved this study. This foundational work will help identify the needs and challenges of key stakeholders in the ED diagnostic process and develop initial design ideas, specifically focusing on sociotechnological ideas for patient-, health care provider–, and system-level interventions for improving patient safety in EDs.

**Results:**

The recruitment of participants for ED health care providers and patients is complete. We are currently preparing for PD sessions. The first results from design sessions with health care providers will be reported in fall 2024.

**Conclusions:**

The study findings will provide unique insights for designing sociotechnological interventions to support ED diagnostic processes. By inviting frontline health care providers and patients into the design process, we anticipate obtaining unique insights into the ED diagnostic process and designing novel sociotechnical interventions to enhance patient safety. Based on this study’s collected data and intervention ideas, we will develop prototypes of multilevel interventions that can be tested and subsequently implemented for patients, health care providers, or hospitals as a system.

**International Registered Report Identifier (IRRID):**

DERR1-10.2196/55357

## Introduction

In emergency departments (EDs), diagnosing and managing patients require highly complex processes involving multiple stakeholder interactions (eg, physicians, nurses, trainees, consultants, caregivers, and patients). A unique aspect of EDs is that various stakeholders make crucial medical decisions in a time-pressured environment. Because of this unique nature, health care providers in EDs face significant challenges in making accurate and timely diagnoses, often causing patient safety issues. While precise error rates are unknown, a conservative estimate of 5% of errors in adults of the 131 million annual ED visits translates to about 7 million cases of ED-based diagnostic errors, with nearly half of them having potential for patient harm [1].

Numerous interventions have supported health care providers’ work and promoted patient safety in clinical settings. For instance, recent artificial intelligence (AI) systems have been proposed and designed to help nurses and physicians in various aspects, such as an AI-assisted autocomplete system [2] for increasing the quality and efficiency of documentation and data entry and an AI-based smartphone app, Face2Gene developed by FDNA Inc [3], for helping health care providers in the diagnostic process of rare congenital disorders. Patient-facing technologies have also been developed, such as an indoor navigation and communication system [4] for estimating patients’ location and helping patients send request messages to health care providers (eg, “Can I have medicine now?”) and a mobile display [5] presenting a digital report on patients’ progress, care plans, and care teams’ work throughout their ED stay. While these technological interventions provide efficiency in health care providers’ cognitive work and patient safety efforts, direct input from patients and health care providers is often overlooked when developing such solutions.

Participatory design (PD) [6] is a design methodology that engages all stakeholders in the design process to create a functional solution that addresses their needs. PD has been used in prior works in health care and medical domains to design technology that meets the specific needs of health care providers. Despite the benefits of PD in designing health technology, there has been limited adoptions of a user-centered PD approach to develop technology interventions for the ED. Østervang et al [7] conducted PD workshops with health care providers and patients to design an ED information system. They presented how the PD approach helps yield insights from ED health care providers and patients to create a more person-centered system. Yet ED patients had limited participation since the workshops were conducted one-on-one, and patients were only asked to provide feedback on intervention ideas developed by health care providers.

This proposed study aims to design ED-based diagnostic error prevention interventions by incorporating key stakeholders’ needs, preferences, and perspectives. Our study will bring key stakeholders together to understand the perspectives of both patients and health care providers and seek to incorporate their input into the design process for future interventions. To involve the stakeholders in the design process, we will deploy a co-design approach using PD methods with patients, caregivers, and clinicians to gather their insights, generate and critique design ideas, and co-design interventions. Through this, we aim to ultimately identify at least 1 intervention for further development at each of the following levels: patient-involved, clinician-focused, and health care system–oriented.

## Methods

### Overview

To accomplish our research goal, we assembled a research team with diverse expertise in emergency medicine, cognitive psychology, systems engineering, informatics, human-computer interaction and design, anthropology, and public health. We are creating a research project group, Improving Diagnosis in Emergency and Acute Care-Learning Laboratory, to investigate ED diagnostic processes and study system vulnerabilities and develop and iteratively test patient-, health care provider–, and system-oriented interventions to mitigate diagnostic error.

This study is part of a larger mixed methods project aiming to identify the challenges of ED health care providers and patients and design multilevel interventions for them. This study has 2 specific parts (Table 1), focusing on developing intervention ideas by inviting ED health care providers and patients to the design process. The study will be conducted in 2 academic medical centers: the University of Michigan and the Mayo Clinic. Participants will be recruited from each center’s ED. We will conduct separate study sessions for ED health care providers and patients. Combining findings from both groups, we will develop multilevel (patient-involved, clinician-focused, and health care system–oriented) intervention ideas to enhance the ED diagnosis process. Ultimately, we will create prototypes of those interventions for future evaluations.

**Table 1:** Overview of the previous and proposed studies. The proposed study has two parts: participatory design with (1) ED^a^ health care providers and (2) ED patients or caregivers to explore their needs and generate design ideas for technological interventions for the ED care process.

	Previous study [8]	Proposed study	
		Part 1	Part 2
Purpose	To understand the current ED care process and preliminarily assess the challenges faced by ED health care providers and patients or caregivers in the diagnostic process	To explore health care providers’ needs and generate design ideas for technological interventions for the ED care process	To explore patients’ or caregivers’ needs and generate design ideas for technological interventions for the ED care process
Research questions	What are the needs and challenges of ED health care providers and patients or caregivers related to the diagnostic process, patient safety, and overall experience during ED care?	How do ED health care providers perceive suggested technological interventions? What are the expected impacts of the intervention?	How do ED patients or caregivers perceive suggested technological interventions? What functions do they expect from the interventions?
Approach	Field observation and interviews	Participatory design	Participatory design
Results or expected outcomes	Scenarios that describe technological interventions to address challenges in the ED process	A ranked list of design ideas that address health care providers’ challenges in the ED process and improve or refine ideas	A ranked list of design ideas that address patients’ and caregivers’ challenges in the ED process and improve or refine ideas

^a^ED: emergency department.

### Idea Generation on ED Care Intervention Design

The findings from our previous study [8] (described in Table 1) using direct observation and participant interviews have helped us identify key needs and challenges health care providers and patients encountered in the ED diagnostic process. For instance, our analysis showed that ED health care providers need a better representation of patients’ statuses to enhance their triage process. They want a system for enhanced collaboration within the care team in the fast-paced ED context. Notably, we identified different challenges stratified by health care provider role (nurses, physicians, and both nurses and physicians), as shown in Textbox 1.

**A list of identified problem categories from the previous study’s emergency department (ED) health care provider interview data. We identified 2 problems from nurses, 4 from physicians, and 4 from both nurses and physicians.**

**Nurses**

- Forgetting to perform patient reassessments due to heavy workloads.
- Difficulty in remembering timely patient reassessment and keeping track of their conditions.

**Physicians**

- Clinicians' high levels of stress can impede decision-making and focus on ED work.
- Physicians’ high cognitive load may interfere with their ED diagnosis work.
- Lack of decision support tools that aid in diagnostic decision-making for increased accuracy.
- Difficulty accessing scattered patient history in a concise and easy-to-read format.

**Both nurses and physicians**

- Insufficient communication between physicians and nurses about orders and next steps in patient care and a lack of electronic health record support for such communication.
- Insufficient communication between physicians and nurses about the patient’s diagnosis and no established opportunity to discuss diagnoses before discharge.
- Lack of notification and information about incoming patients with critical care needs.
- Acuity level differs between nurses and physicians. Physicians sometimes have to reassign acuity levels mentally.

For patients, one of the major challenges was limited time spent with the caregiver team, which was perceived as the absence or lack of support. The patients often received less attention, care, or information about the ED process than expected or needed. Based on these challenges, we identified 7 problem categories that previous study patients experienced (described in Textbox 2).

**A list of 7 identified problem categories from the previous study’s patient and caregiver interview data.**

**Waiting room general challenges**

- Patients feel forgotten in the waiting room because health care providers do not check on them.
- Patients are left in pain or experience other unresolved symptoms in the waiting room.

**Overcrowding challenges**

- General overcrowding problems (eg, there are patients with mild symptoms who make emergency department [ED] more crowded).

**Information presentation and overload challenges**

- Patients are not sufficiently informed of the ED process during the visit (lack of ED process literacy).
- Test results can be confusing for patients to understand because of the use of medical terminology.

**Information sharing challenges**

- Patients often feel that there are errors in the doctor’s notes (which are different from what was discussed during the visit).
- Health care providers at hospitals sometimes wait for information transfer from the patient’s original hospital, which can take time.

**Communication challenges**

- Patients find it hard to recount all personal medical details in the ED because they are sick, stressed, or unaware.
- Patients sometimes have trouble communicating with health care providers because of challenges such as being a nonnative English speaker.

**Contagious disease transfer in ED**

- Crowded waiting rooms can have many sick patients in close proximity, which makes patients concerned.
- Contact with surfaces can cause disease transfer.

**Discharge and postvisit challenge**

- Lack of postvisit resources for patients.
- Patients feel that they have been wrongly discharged.

We have developed various initial ideas for ED care interventions based on the identified challenges that the ED health care providers and patients faced in the ED diagnostic process. These design ideas describe technological solutions that can serve as probes to identify the challenges and needs of ED health care providers and patients. For health care providers, the design ideas include automated tools to support health care providers in triaging patients, visualization tools to help monitor patients’ conditions, wearable devices to track and manage health care providers’ stress, and communication tools for the ED care team. For patients or caregivers, intervention idea categories include robots to alleviate patient anxiety in the waiting room, visualization tools to inform them about the procedures, and systems to enhance their communication with health care providers. Based on these design ideas, we will select novel ideas to develop them into intervention scenarios. Those scenarios describe how each intervention can resolve a specific challenge for ED health care providers or patients. The scenarios will be used to facilitate discussions in the PD sessions with ED health care providers (part 1) and patients or caregivers (part 2).

### Part 1: PD With ED Health Care Providers

#### Sampling, Eligibility, and Recruitment of ED Health Care Providers

We will recruit ED health care providers with different roles (eg, emergency medicine and pediatric emergency medicine–trained physicians, fellow physicians, and nurses) and explore how different roles engage in the ED care process. Eligible health care providers will be ED physicians and nurses who have worked a minimum of a year in the ED setting. We will recruit 3-4 health care providers for each session. In total, 4-5 sessions are planned, and additional sessions may be conducted if data saturation is not reached. The health care providers will be recruited in 2 medical centers, as described earlier. A study coordinator will recruit health care providers through email or in person on the day of a shift. Once they express their interest in participating in our study, we will obtain electronic informed consent via email before each session. We will offer a US $100 gift card to health care providers for their participation in a design session. All PD sessions will be audio-recorded and transcribed for data analysis.

#### Data Collection Instruments

**Figure 1:**
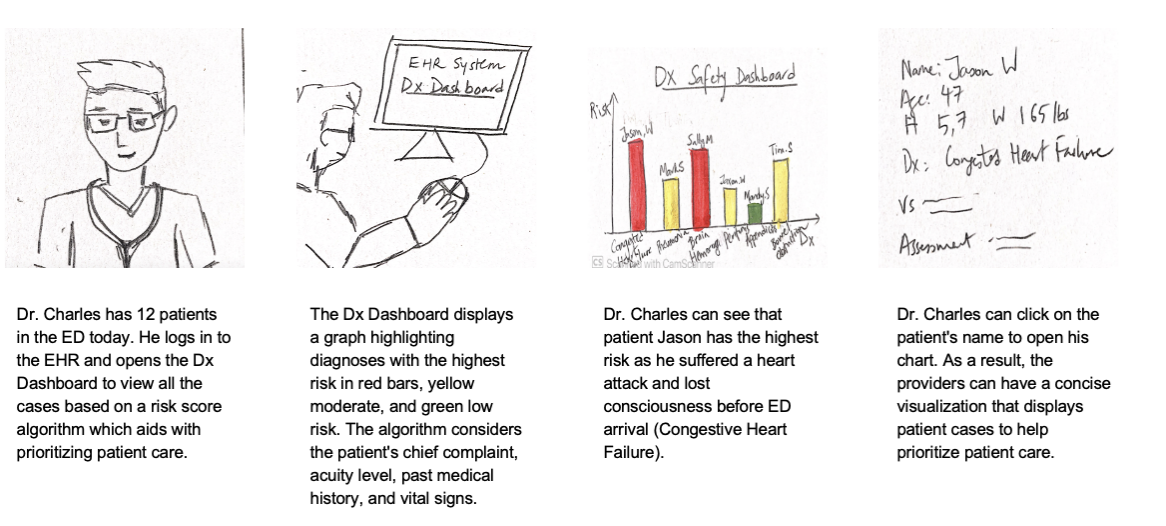
A sample storyboard presenting our design idea from the session with ED health care providers: an artificial intelligence–based dashboard that visualizes patients’ risk levels so that health care providers can prioritize patient care more efficiently. ED: emergency department.

Before conducting design sessions, we will create storyboards that describe our design ideas about potential technological interventions and their use scenarios. The storyboards will be based on health care providers’ challenges in seeking and using the information necessary for the ED diagnostic process, as shown in Textbox 1. For instance, a storyboard will describe how an AI-based dashboard can compute the patient care priority based on the risk factors of each ED patient and visualize the priority with simple graphs (Figure 1).

#### Activity 1: Design Idea Critique With Health Care Providers

Each PD session will involve 3 or 4 ED health care providers with the same role (eg, physician or nurse) from the same medical organization to avoid any impact of power dynamics between different roles [9]. Depending on the health care providers’ availability, each session will be conducted in person in a private conference room at one of the medical organizations or remotely through a web-based platform (Zoom; Zoom Video Communications). Each PD session will have two major activities: (1) design idea critique and (2) co-design activity. For the critique activity, we will use storyboards to present the intervention ideas to participants. Participants will review the storyboards and have a group discussion about the appropriateness, feasibility, and applicability of each intervention idea and features that should be added. We will prepare follow-up questions to facilitate group discussions (eg, “How do you want the AI system to present the emergency severity index about patients?”). Besides those questions, participants can also freely share their thoughts and perspectives on each storyboard.

#### Activity 2: Co-Design With Health Care Providers

In the second activity, among the storyboards they discussed, participants will be asked to choose the most helpful intervention idea and improve or modify it individually. Then, they will share their ideas with other participants and reflect on the ideas together as a group. For the in-person sessions, we will prepare drawing materials (eg, pencils, colored pencils, and paper) to make the co-design activity more engaging and effective. For the remote sessions, we will ask participants to prepare their drawing materials in advance (at least a pencil and a piece of paper). As a group, all participants will combine their ideas into 1 intervention idea that everybody agrees upon. All sessions will be audio- and video-recorded for data analysis. Participants’ drawings will be collected after design sessions. For the remote sessions, participants will be asked to take a picture of their design and send it to one of the researchers, who will share the design with all the participants.

#### Data Analysis

We will use a thematic analysis approach [10] to analyze the transcripts from design sessions. Thematic analysis is a methodical tool for discovering repeating themes in the text by finding common topics or ideas that keep coming up. It represents the structured outcome of a collaborative brainstorming session. Adopting the thematic analysis, the research team members will code the transcripts separately first. We will also analyze and code our observation notes from each session and participants’ designs from the activity. Photos and recorded videos will capture detailed participant interactions during each session. Then, the researchers will combine the codes, compare their codes, and identify recurring themes. The research team will discuss the themes to identify which aspects of each intervention participants like or dislike. The research team will also analyze potential differences between 2 sites or roles (eg, physician vs nurse). This analysis will help us better understand participants’ nuanced technology preferences and provide insights into how to balance different perspectives in designing future technologies to support ED health care providers.

### Part 2: PD With ED Patients or Caregivers

#### Sampling, Eligibility, and Recruitment of Patients or Caregivers

We will recruit ED patients and caregivers from the adult and pediatric EDs at the University of Michigan. Eligible participants are those 18 years or older or whose caregivers have visited an ED (either pediatric or adult) in the last 6 weeks. We will recruit 4-6 patients or caregivers for each session. The expected number of sessions will be 4-5, but we may conduct more sessions if data saturation is not reached. A few well-trained researchers will visit the ED and introduce the study to patients in the waiting room. We will collect their contact information for follow-up scheduling once they express interest in participating in our study. We will contact interested patients and ask them to complete a digital poll to provide their availability. This information will be used to find a suitable time to conduct a session with 4-6 participants. We will also post a flyer with a study description and our contact information at the hospital. Our research team staff will contact the eligible participants through calls, SMS text messages, or emails. Patients and caregivers will receive a US $100 gift card for participating in a design session. We will obtain electronic informed consent from all patient or caregiver participants by emailing the form before each session. If they do not fill out the digital form, we will obtain a paper copy of the consent form and ask them to sign it at the beginning of the in-person session. All PD sessions will be audio-recorded and transcribed for data analysis.

#### Data Collection Instruments

**Figure 2:**
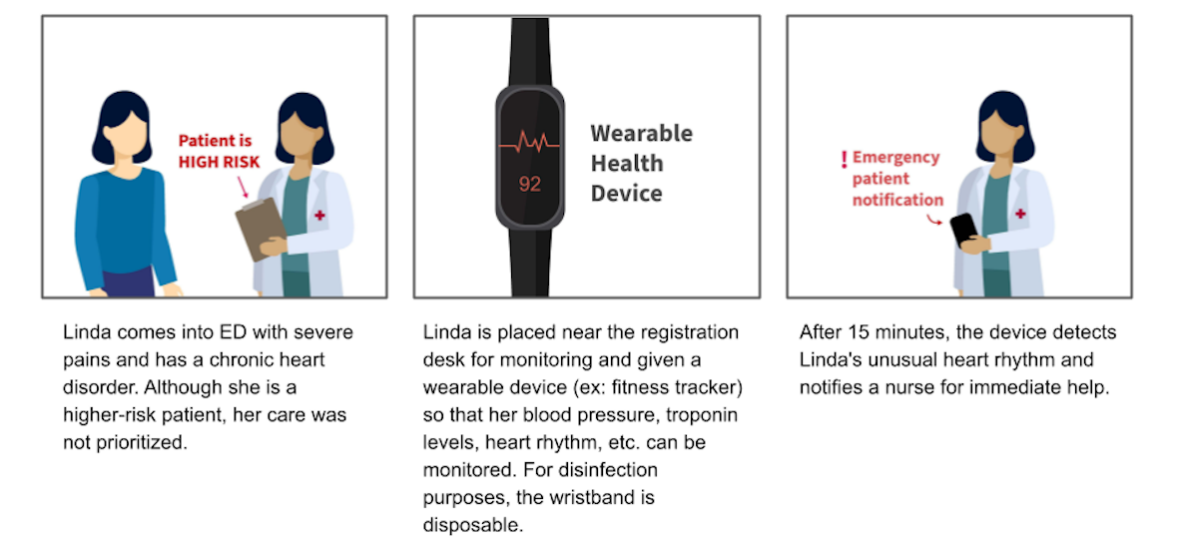
A sample storyboard presenting our design idea from the session with patients and caregivers: a wearable device for ED patients in a waiting room that tracks patients’ real-time conditions and notifies health care providers if the patients need immediate help. ED: emergency department.

Like part 1, we will create storyboards describing our design ideas about potential technological interventions and their use scenarios. The storyboards will be based on patients’ challenges in seeking and using the information necessary for the ED diagnostic process, as shown in Textbox 2. For instance, a storyboard will present how a wearable device for ED patients in a waiting room can track patients’ real-time conditions and notify health care providers if a patient need immediate attention (Figure 2).

**Figure 3:**
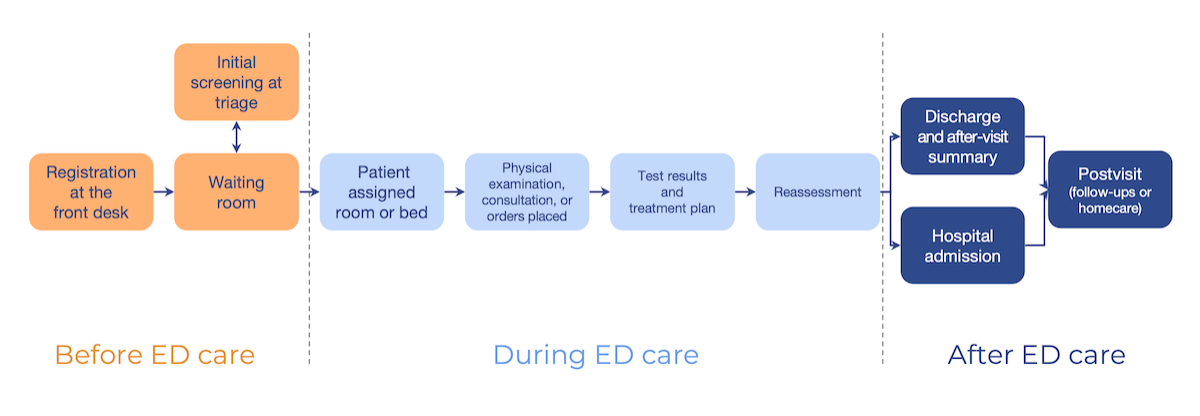
An ED care process timeline that illustrates the steps before, during, and after ED care. We created this timeline based on prior studies. ED: emergency department.

For patients, we will also provide a paper copy of a typical timeline of the ED care process (Figure 3). We created this timeline based on our previous study [8] and prior work on the ED care framework [11]. Unlike health care providers, patients may need more information about the step-by-step ED care process. Thus, the timeline will help patients understand the structure of the process and remind them of their own recent ED experiences. We will ask participants to share the challenges they experience throughout the timeline.

#### Activity 1: Design Idea Critique With Patients and Caregivers

Each session will involve 4-6 patients or caregivers. Caregivers will include patients’ family members or friends who visited an ED with the patient. The sessions will be conducted in person in a private room at one of the medical organizations, following a format similar to the activities described in “PD with ED health care providers.” Participants will also be given a handbook that includes a paper copy of each storyboard, the ED care process timeline, and additional pages for their notes. Before the main activity, we will begin the sessions with patients by introducing the ED process timeline so they can easily remember the experiences and challenges they faced during their recent ED visits.

Similar to part 1, the first activity will be critiquing design ideas. We will use storyboards to present the intervention ideas to participants. Participants will review the storyboards and have a group discussion about the appropriateness, feasibility, and applicability of each intervention idea and additional features that should be added. We will prepare follow-up questions to facilitate group discussions for each intervention idea (eg, “What types of information can the chatbot provide?”). In addition, we will verify the problem described in each storyboard by asking participants specific questions (eg, “Have you ever felt that providers needed to explain more about test results or processes but were too busy?”). Besides those questions, participants can also freely share their thoughts, perspectives, and experiences related to the problem described in each storyboard. During this activity, participants can take notes on their handbook, which has a paper copy of each intervention.

#### Activity 2: Co-Design With Patients and Caregivers

In the second part of the PD workshop, participants will choose their favorite storyboards based on their ED experiences and knowledge, and they will share their reasoning with the group in order to choose one storyboard as a group. Then, all participants will design their ideas as a group to improve or modify the intervention idea. We will prepare drawing materials (eg, pencils, colored pencils, and paper) and whiteboards for the design activity. The participants will then present their ideas to the research team members, and the researchers will ask follow-up questions to probe the group’s reasoning for each design decision. All sessions will be audio- and video-recorded for data analysis. Participants’ drawings will be collected after the design sessions.

#### Data Analysis

Like part 1, we will use a thematic analysis approach [10] to analyze transcripts, participant notes, and observation notes from the sessions. To circumvent biases of subjective interpretation of the qualitative data, at least 2 research team members will code the transcripts separately. Photos and recorded videos will also capture detailed participant interactions during each session. Then, the research team members will combine the codes, compare their codes, and identify recurring themes. The team will discuss the themes related to patient needs, challenges, and aspects of each intervention. This analysis will help us better understand participants’ nuanced intervention ideas and provide insights into possible future technologies to improve patients’ experience of ED visits.

### Prototype Development

By combining data collected from both health care providers and patients, we will explore potential intervention ideas at 3 levels: patient-focused, health care provider–focused, and system-focused interventions. This co-development process with participants will initially allow user input and flexibility but ultimately lead to a concrete version of a technological intervention that provides effective support to patients, ED health care providers, and hospitals as a system.

Drawing on the results from the analysis, we will develop prototypes of the top intervention ideas from participants, using an iterative approach. The iterative approach has been used in various design projects and proven its effectiveness in improving the quality of prototype designs [12-14]. The iterative process will have 3 main steps. First, we will develop a series of low-fidelity prototypes based on the 3-level interventions. Second, we will conduct usability evaluations with health care providers and patients or caregivers. Third, we will revise the prototypes based on the feedback and return to the second step. Through this iterative process, we will identify the most feasible and helpful intervention ideas for future implementation.

### Ethical Considerations

We will comply with the following ethical considerations. First, we will get consent from all participants before the study sessions as described in the multisite institutional review board protocol approved by the University of Michigan (HUM00156261). Second, we ensure that the transcription data is deidentified, and participants’ faces in photos or videos will be blurred. All collected data will be stored in a secure storage facility that requires University of Michigan accounts. Third, all researchers will complete research compliance training on best practices and ethical considerations of interacting with health care providers and patients, such as those offered by the Collaborative Institutional Training Initiative or the Program for the Education and Evaluation in Responsible Conduct of Research.

## Results

The recruitment of participants for ED health care providers and patients is complete. We are currently preparing for PD sessions. The results from design sessions with health care providers and patients will be reported separately in fall 2024. The results of this study will be disseminated through journal publications and presentations at national and international meetings.

## Discussion

### Anticipated Findings

PD is a useful methodology for incorporating stakeholders’ needs in the early stage of the intervention development process by verifying the overlap between the needs a design research team observed during fieldwork and the needs target users perceive in their own lives as well as their preferences for new technology. Specifically focusing on the ED care contexts, the proposed study will contribute to extending the understanding of the challenges experienced by ED health care providers and patients and their expectations for interventions to improve the diagnostic process and patient safety.

The proposed study has 3 anticipated contributions. First, the study findings will reflect the nuanced needs of patients and health care providers during PD sessions. These user ideas will help us and other researchers to understand their needs and the barriers encountered in the ED decision-making process, which will inform the design of technological interventions to meet these needs. Second, recruitment of participants from 2 sites with varying characteristics may provide diverse perspectives and broader intervention insights. The findings may show differences between participants from different sites, which may provide design insights for mitigating conflicting user needs when developing technological interventions for the ED decision-making process. Third, this study will present how the PD approach can be used to develop health technologies that support the diagnostic process in the ED, thereby extending findings from previous work on PD in hospital settings by focusing on the distinction between the needs of the ED health care providers and those of the patients. A few prior studies conducted PD in hospital settings. For instance, Kusunoki et al [15] conducted PD workshops with trauma team members to understand the different needs of awareness support among the various roles of team members and identify concrete design strategies to manage the differences in their awareness needs. Pollack et al [16] organized a design session with 11 clinicians to develop a clinical information tool using PD techniques. Based on the session’s findings, the authors identified benefits (eg, a high level of domain knowledge can be used to anticipate how design ideas can be applied to clinical processes and workflow) and potential challenges (eg, power dynamics between physicians). The authors also outlined guiding principles for implementing these methods in health care organizations interested in advancing their use of health information technology. These prior studies have presented how PD is helpful and effective for designing human-centered technology in health care settings. Along with these previous studies, this proposed study will provide insights for conducting PD with multiple stakeholders, particularly extending the involvement of ED patients whose roles were often limited [7]. In addition, the proposed study findings will provide rich insights into design implications for technology to support decision-making in the ED diagnostic process by incorporating perspectives and ideas from patients and health care providers.

### Limitations

This study has several limitations. First, given its qualitative nature, the findings are specific to the participating sites, as health care providers and patients from other EDs may face different challenges. Yet, we anticipate our findings will contribute to identifying such challenges and potential interventions to address the challenges through a PD approach. Second, even though we leveraged evidence from preliminary interview data, some intervention ideas we developed are novel technologies, with which some participants may have no prior experience (eg, tabletop robots). Thus, it may be challenging for the participants to contextualize using these nonexistent technologies without a tangible prototype.

### Conclusions

The study findings will provide unique insights into designing sociotechnological interventions to support ED diagnostic processes. Inviting ED health care providers and patients into the design process will facilitate the design of sociotechnological interventions to address the specific needs of ED health care providers and patients. Finally, we will develop prototypes based on this study’s findings and intervention ideas, which will be developed iteratively in future evaluation studies.

## Appendix

Multimedia Appendix 1 Peer review report from the Agency for Healthcare Research and Quality (AHRQ).

## Abbreviations

AI: artificial intelligence
ED: emergency department
PD: participatory design

## Data Availability

The data sets generated in this study are available from the corresponding author upon reasonable request.
