# Supplementary material for: Identifying Interventions to Improve Diagnostic Safety in Emergency Departments: Protocol for a Participatory Design Study
Source: JMIR Res Protoc. 2024 Jun 21;13:e55357. doi: 10.2196/55357 (PMC11226926; doi:10.2196/55357)
Supplement: Multimedia Appendix 1 [file resprot_v13i1e55357_app1.pdf]

RESUME AND SUMMARY OF DISCUSSION: This grant application, submitted by Prashant Mahajan from the University of Michigan at Ann Arbor and entitled, "*Improving Diagnosis in Emergency and Acute Care: A Learning Laboratory (IDEA-LL),*" responds to RFA-HS-18-001 Patient Safety Learning Laboratories: Pursuing Safety in Diagnosis and Treatment at the Intersection of Design, Systems Engineering, and Health Services Research Grant (R18). The application proposes to create "Improving Diagnosis in Emergency and Acute care - Learning Laboratory" (IDEA- LL), a multi-site program for diagnostic safety surveillance and intervention. The study aims to develop and use innovative systems engineering (SE)-based approaches for better measurement and reduction of diagnostic error in the emergency department (ED). The reviewers noted several strengths in the application. The proposed study is significant because there are approximately 7 million cases of diagnostic errors in U.S. EDs annually. The proposed study builds on prior AHRQ-funded research and links to current research collaborations. The team is well suited for the project with complementary and integrated expertise in clinical, human factors, and social science methods. The PI has a strong record of ED safety and healthcare quality management. The PI and Co-PI have worked together on other studies. IDEA-LL will be the first to study the diagnostic process comprehensively from the pre-hospital phase until patient disposition. Implementation of an EHR-based error risk prediction tool to achieve the study's aims is innovative. IDEA-LL will use actionable, patient-centered data obtained from frontlines of care and electronic health records (EHRs). IDEA-LL will use mixed methods to design, implement, and evaluate interventions to improve diagnostic safety. The collaborating centers, organizations, and institutions are well established and supportive of the proposed study. The participating centers appear to have sufficient number of clinicians and patient visits appropriate for the proposed subject recruitment. Nevertheless, the reviewers identified some weaknesses in the application. Reviewers commented that the implementation phase and planned interventions lack details. For example, the application does not fully describe specific anticipated interventions. It is unclear how the interventions will be developed and whether the final interventions will be evaluated separately or together. The evaluation plan lacks details on the target sample population, data collection and analysis, and measurement of the interventions' effectiveness. Reviewers were concerned that there are insufficient hours to collect data and to develop, implement and evaluate interventions. Reviewers also expressed concern that the recruitment plan creates the potential for selection bias. Overall, the reviewers rated this application from "Excellent to Outstanding" range with a level of "High Impact".

DESCRIPTION (provided by applicant): Diagnostic decision-making is a highly complex cognitive process involving uncertainty, which makes it susceptible to errors. Clinicians working in emergency departments (EDs) are particularly vulnerable to making diagnostic errors because of time-pressured decision-making in chaotic environments. There are ~ 141 million annual ED visits in the US. A conservative estimate of a 5% diagnostic errors in adults translates into ~ 7 million cases of diagnostic errors in the ED, with nearly half with potential for patient harm. Diagnostic errors result from a complex interplay between various patient (health literacy, presenting complaint, complexity, etc.), provider/care-team (cognitive load on providers, information gathering/synthesis, etc.) and systems (health information technology, crowding, interruptions, etc.) factors. To reduce diagnostic errors in the ED, we must use methods that illustrate the dynamics of human-system interaction during diagnostic process. Our goal is to create "Improving Diagnosis in Emergency and Acute care - Learning Laboratory" (IDEA-LL), a novel program for diagnostic safety surveillance and intervention using actionable, patient-centered data obtained from both frontlines of care and electronic health records (EHRs). IDEA-LL will use multidisciplinary approaches to design, implement and evaluate interventions to improve diagnostic safety. The investigative team, led by a unique physician-engineer partnership, will form a transdisciplinary environment of clinicians, nurses, patients, engineers, informaticians and designers as an integral aspect of the learning laboratory to address both pediatric and adult emergency care in academic and community EDs. In Aim 1 (identify), to understand the detailed process of diagnostic decision-making and identifying potential factors that lead to diagnostic errors we propose an iterative process using mixed methods-grounded theory, i.e. combining qualitative (participant observations, in-depth participant interviews) and mining of historical data. We will use direct in-situ observations at two

academic and two community EDs to map the entire diagnostic process. We will supplement the observations by stakeholder interviews with ED clinicians and patients to obtain perspectives and perception on vulnerabilities in the diagnostic process. We will supplement prospective observation by conducting a retrospective analysis of medical records that were trigger positive to compare with control records to assess potentially contributing variables. In Aim 2 (design and development), using consensus methods we will develop a comprehensive list of patient, provider/care-team and system level contributory factors and identify interventions to be studied. After ranking potential interventions, using human-centered design principles with input from human-factors engineers, we will isolate patient, provider/care-team and system-focused intervention for iterative testing and deployment for efficacy testing at the four EDs. In Aim 3 (implementation and impact), we will test for effectiveness and impact of the interventions at the 4 EDs using mixed methods i.e. quantitative and qualitative measures.

PUBLIC HEALTH RELEVANCE: Diagnostic decision-making is a highly complex cognitive process involving uncertainty, making it susceptible to errors. Clinicians working in emergency departments (EDs) are particularly vulnerable to making diagnostic errors due to time-pressured decision-making in a hectic environment. Traditional approaches to study errors and address patient safety are retrospective case-by-case root-cause analysis, which are often reactive placing the burden on providers and sometimes on patients. To address these issues, we will use data-driven, human-centric and action-oriented systems engineering principles to study diagnostic errors, design and implement interventions that can improve care delivery at large. Our goal is to develop and use innovative systems engineering-based approaches for better measurement and reduction of diagnostic error in the ED. Our novel approach, “Improving Diagnosis in Emergency and Acute Care: A Learning Laboratory (IDEA-LL),” will collect and analyze data on diagnostic errors from both frontlines of care and EHRs, design, implement and evaluate interventions to improve diagnostic quality and safety.

CRITIQUE NOTE: The written critiques of individual reviewers are provided in essentially unedited form below. These critiques were prepared prior to the meeting and may not have been revised afterwards. The “Resume and Summary of Discussion” above summarizes the final opinions of the committee.

CRITIQUE 1

Significance:	1
Investigator(s):	2
Innovation:	1
Approach:	3
Environment:	1

Overall Impact:
Strengths

- “Improving Diagnosis in Emergency and Acute care - Learning Laboratory” (IDEA-LL) is a multicenter, patient safety-learning lab that will develop and use innovative systems engineering-based approaches for understanding and reducing diagnostic error in the ED.
- Builds on a recently adapted ED- specific conceptual framework and uses novel approaches informed by team’s earlier work, including newly developed triggers, to measure the risk of errors. Aligns three conceptual approaches, namely a modified NASEM framework for the diagnostic process contextualized to the ED1, the missed opportunity approach to contextualize error, and the learning health systems model of data- performance-knowledge.
- Members of our team have performed foundational AHRQ-funded work in (a) defining opportunities to improve diagnosis in the ED,1 (b) proposing a socio-technical model for diagnostic safety (Safer Dx framework), and (c) developing an ED-based conceptual framework

of the diagnostic process. The current RFA provides an opportunity to use systems engineering principles to validate the work in real world ED settings.

Weaknesses

- The project will identify and evaluate the impact of at least one patient-focused intervention (e.g. re- engineered approach to engage patients at discharge for certain at-risk conditions), one provider-focused intervention (e.g. diagnostic huddle for a potential never-miss condition), and one systems-focused intervention (e.g. decision support tool for high-risk situations). It is not clear that the final interventions will be evaluated as a system as opposed to three discreet interventions. i.e., what are the individual and combined impacts?

1. Significance:

Strengths

- Addresses a significant problem: ~7 million diagnostic errors in EDs, of which more than half are likely preventable.
- Builds on the teams' recent foundational work, the project will prospectively map the anatomy of the entire diagnostic process, from the pre-hospital setting to patient disposition from the ED, and identify areas of risk. The prospective data will be augmented with information obtained from safety events identified through a review of EHR "triggers".
- By understanding the complex interplay between various patient (health literacy, presenting complaint, complexity, etc.), provider/care-team (cognitive load on providers, information gathering/synthesis, etc.) and systems (health information technology, crowding, interruptions, etc.) factors the lab will develop system-responsive interventions that will reduce diagnostic errors in the ER.

2. Investigator(s):

Strengths

- The team is well suited for the project with complementary and integrated expertise in clinical, human factors and social science methods.
- The team is led by an appropriate physician-engineer partnership, with sufficient FTE in each year.
- The team has complementary skills and includes patient representatives as well as experts in clinical care, systems engineering, patient safety, informatics, mixed methods research, human-centered healthcare design, statistical monitoring of complex care delivery systems, learning health systems.
- The leadership approach, governance and organizational structure appropriate for the project.

Weaknesses

- Small FTEs (1.8 months each) for PI/Co-PI and Co-Is. It does not appear that there are personnel with enough time to collect data, develop, implement and evaluate interventions.

3. Innovation:

Strengths

- Builds upon previous work to address patient-level, provider/team- level, and systems-level factors that influence diagnostic safety and uses a multidisciplinary approach to understand and reduce diagnostic errors.
- IDEA-LL will be the first to study the diagnostic process comprehensively from the pre- hospital phase until patient disposition.

4. Approach:

Strengths

- The approach adequately describes the 5 phases. Uses systems engineering approaches to identify factors contributing to ED diagnostic error.

- Multi-method approach to provider, clinician, patient stakeholder involvement, participatory design, data mining, machine learning, ethnographic study using direct in situ observation, prospective pre and post-intervention observational study.
- The project will identify and evaluate the impact of at least one patient-focused intervention (e.g. re- engineered approach to engage patients at discharge for certain at-risk conditions), one provider-focused intervention (e.g. diagnostic huddle for a potential never-miss condition), and one systems-focused intervention (e.g. decision support tool for high-risk situations).
- Two PIs of this proposal are already collaborating on an AHRQ supported project (1R01HS024953) that led to development of a consensus-based conceptual framework for the diagnostic process in the ED.
- Implementation and evaluation in 2 academic and 2 community EDs to demonstrate generalizability representing urban, suburban and rural.

Weaknesses

Small FTEs for PI/Co-PI and Co-Is. It does not appear that there are personnel with enough time to collect data, develop, implement and evaluate interventions. Specifically, it is not clear who will perform the following:

- Aims 1.1 & 1.2: Mixed methods - 1) ethnographic study using direct in situ observations at the 4 EDs with trained observers conducting observations of the ED providers, patients, and their caregivers; 2) ED diagnostic process mapping and 3) provider/patient/stakeholder interviews.
- Aim 2: workshops to co-design ED-based interventions with input from patients and ED providers.
- Aim 3: Mixed-method qual and quant prospective pre- and post-intervention observational study to test the feasibility of the intervention at the four EDs evaluation to demonstrate the impact of interventions on risk based quantitative outcomes.

- The implementation phase is not well described. How will it be evaluated? What are the outcome measures?
- It is not clear that the final interventions will be evaluated as a system as opposed to three discreet interventions. i.e., what are the individual and combined impacts?

5. Environment:

Strengths

- The collaborating centers, organizations and institutions are well established, have a history of collaboration and have provided ample evidence of the necessary enthusiasm, space, equipment and facilities to support the project.

Protections for Human Subjects

- Acceptable.

Inclusion of Women and Minority Subjects:

- Acceptable.
- Reasonable given the patient population.

Inclusion of AHRQ Priority Populations:

- Acceptable.
- Reasonable given the patient population.

Degree of Responsiveness:

- Acceptable.
- The topic and transdisciplinary team are highly responsive to the purpose and objectives of the FoA. It applies scientific insights to understand the elements that influence health outcomes;

models the relationships between those elements; and will develop and implement systems based on systems engineering that includes a systematic study of all related factors to detect and decrease diagnostic errors.

Budget and Period of Support:
Strengths
- The overall budget and timeframe are reasonable.

Weaknesses
- It is unclear who will perform the bulk of the data collection in Aims 1, 2, and 3.

CRITIQUE 2

Significance:	2
Investigator(s):	2
Innovation:	2
Approach:	3
Environment:	1

Overall Impact: The aim of this project is to create a novel program for diagnostic safety surveillance using frontline observation, interviews, and mining of EHR data to understand diagnostic decision making and identify potential factors leading to diagnostic errors. A retrospective analysis of EHR that are "trigger positive" will be compared to control "trigger negative" records. Using consensus methods, a comprehensive list of contributory factors and potential interventions will be identified. Patient, provider/care team, and system focused interventions will be selected for iterative testing and efficacy will be deployed at 4 EDs. The interventions will be tested for efficacy and impact.
Strengths
- Builds on prior AHRQ research and linked to current research collaborations.
- Uses mixed methods to include EHR analysis and qualitative component.
- Identifying ED diagnostic error prevention interventions has the potential to decrease diagnostic errors and improve patient safety in the ED.
- Multi-institution, multi-site in both academic and community EDs.

Weaknesses
- Additional studies will be needed to determine effectiveness of interventions.
- Anticipated recruitment difficulties of patients and providers leading to potential selection bias.

1. Significance:
Strengths
- Has the potential to identify "triggers" associated with diagnostic error to develop an ED decision support system that is EHR based to serve as a risk prediction tool.
- Has the potential to reduce diagnostic errors, costs associated with them.

Weaknesses
- They are only going to identify one patient-related, team-related, and systems-related intervention, limiting the understanding of the potential impact.
- Approach to and implementation of final interventions lacks specificity and detail.

2. Investigator(s):
Strengths
- Investigators are well-qualified and with diverse experiences. PI has prior AHRQ funding related to diagnostic decision making. The team has expertise in diagnostic decision-making, informatics, systems design and engineering that is well-suited to this project. Additionally, the PI and Co-PI have prior experience working together.

Weaknesses

- None noted.

3. Innovation:

Strengths

- The project builds upon prior work on poorly-understood diagnostic errors. Implementation of an EHR-based error risk prediction tool leading to the design and develop ED-based diagnostic error prevention strategies is innovative.

4. Approach: Aim 1: Use systems engineering approaches to identify factors contributing to ED diagnostic error.
1.1 Perform prospective ED observation in situ to map the anatomy of the diagnostic process.
1.2 Conduct interviews with key stakeholders including frontline ED staff and patients to identify vulnerabilities of the diagnostic process.
1.3 Use data mining/machine learning to compare an at-risk, trigger-positive sample to trigger-negative charts to identify various patient, provider/care-team and systems factors that influence diagnostic safety.

Aim 2: Design and develop ED-based diagnostic error prevention interventions.
2.1 Use participatory design with patients and ED clinicians to generate intervention design ideas and to identify at least one patient, one provider/care-team, and one system-focused intervention for development, i.e. a “three-pronged intervention approach”.
2.2 Use human-centered design to develop an ED decision support system (ED-DSS), an EHR-based, dynamic, diagnostic error risk prediction tool. Building on the empirical understandings and insights obtained through our ethnographic study and systems engineering approach (Aim1), our second phase of this research project is intervening through design, guided by human/user-centered design principles. We will initiate workshops where we will co-design ED-based interventions with input from patients and ED providers. We will identify design needs for users, generate design features, and develop mock-ups.

Aim 3: Implement and evaluate the impact of interventions on diagnostic error risk reduction in EDs.
3.1 Pilot interventions in 2 academic EDs and 2 community EDs to study feasibility and demonstrate efficacy.
3.2 Perform a mixed method evaluation to demonstrate the impact of interventions on risk based quantitative outcomes (e.g. reduction of diagnostic errors in trigger positive EHRs) and qualitative outcomes (e.g. improvement in diagnostic safety culture) in the two academic and two community EDs.

Strengths

- Building on prior AHRQ funded research on diagnostic error.
- Use of mixed-methods (patient interviews, provider interviews, data-mining/machine learning in EHR) to develop error prevention strategies.
- Incorporation of two institutions in both an academic and community setting.
- Inclusion of pediatric patients.

Weaknesses

- Authors note that prior work has highlighted the importance of the prehospital phase, but I cannot identify any prehospital component in this study.
- While it is a strength to include pediatric patients, it is unclear how or if pediatric errors will rise to inclusion since they constitute a smaller number of total patients and are not included as a separate ED site, though it seems that both Michigan and Mayo have separate pediatric EDs.
- The error risk prediction tool example provided gives an example of how diagnostic error risks decreased over time and with additional testing. First, it is not clear how this tool will be used. Will it be used in real-time to predict diagnostic uncertainty or offer alternative steps for clinical

decision making? If it is only predicting risks, attention should be noted regarding impact on length of stay in the ED, costs, and unnecessary testing.
- Additional details regarding selection of final interventions and evaluation should be provided for improved understanding.

5. Environment:
Strengths
- Excellent and supportive environment.
- Appropriate resources.

Protections for Human Subjects
- Acceptable.
- They have outlines a full set of safeguards they intend to protect human subjects.

Inclusion of Women and Minority Subjects:
- Acceptable.
- All patients will be included in data review. There is an opportunity to define a representative approach to recruitment of patients and providers for the qualitative component of the study.

Inclusion of AHRQ Priority Populations:
- Acceptable:
- All populations are included in this study.

Degree of Responsiveness:
- Responsive to the FOA.

Budget and Period of Support:
- Acceptable, but travel budget seems high.

CRITIQUE 3

Significance:	1
Investigator(s):	1
Innovation:	1
Approach:	3
Environment:	1

Overall Impact:
Strengths
- The proposal seeks to study diagnostic errors in ED, which has not been well defined, measured, and intervened. Due to the unique ED setting, diagnostic errors can make serious harms to the patients.
- The team brings in multiple innovations supported by its prior work, raising the chance of success of the proposed work.
- The proposed approach leverages both prospective and retrospective methods to make use of stakeholder participation and advanced data analytics techniques in the analysis of diagnostics errors and development of prediction/intervention algorithms.
- The team has strong, multidisciplinary, and complementary expertise and collaboration history for the project success.

Weaknesses

- The approaches related to the development and implementation of interventions lack details. For example, anticipated interventions are not clearly known, although several examples are provided [C.5]; explanations related to measurement strategy [C.5.b.2], target sample population [C.5.b.3], and data collection/analysis [C.5.b.5] are quite high level and missing details.

1. Significance:
Strengths
- Diagnostic errors in ED can incur significant harm to patients, but has not been defined, measured, and intervened properly – new approaches to study diagnostic errors in ED is a significant requirement to reduce their occurrence.

2. Investigator(s):
Strengths
- The PI has a solid track record of ED safety and healthcare quality management. Further, the PI formed a strong team of complementary expertise required to conduct proposed work: ED, diagnostic safety, systems engineering, ED clinicians, design, and healthcare informatics.

3. Innovation:
Strengths
- The team brings in multiple innovative tools it has developed to date, which enables formal system engineering investigation of the proposed work [B.1-B.5].

4. Approach:
Strengths
- The approach closely adheres to the 5-step systems engineering methodology with brain storming and rapid prototyping.
- The approach is logical – first to identify sources of diagnostic errors, then develop novel interventions, then implement and test them.
- The use of both prospective and retrospective strategies may enable seamless integration of qualitative and quantitative information into the analysis and development of errors and interventions.
- Some analysis strategies are explained very thoroughly (e.g., how to analyze observation and HER-based data collected in Aim 1).

Weaknesses
- It is not clear how and what kind of interventions will be developed and how they will be implemented [C.5].
- Handling of pre-hospital information, which has been identified to be important by the team, has not been included in the approach (at least explicitly).

5. Environment:
Strengths
- The participating centers appear to have sufficient number of clinicians and patient visits appropriate for the proposed subject recruitment [C.2].

Protections for Human Subjects
- Acceptable.

Inclusion of Women and Minority Subjects:
- Acceptable.

Inclusion of AHRQ Priority Populations:
- Acceptable.

Degree of Responsiveness:
- Acceptable.
- The proposal addresses an important patient safety issue.
- The proposal uses 5-step systems engineering methodology.

Budget and Period of Support:
- Nothing noted.

THE FOLLOWING SECTIONS WERE PREPARED BY THE SCIENTIFIC REVIEW OFFICER TO SUMMARIZE THE OUTCOME OF DISCUSSIONS OF THE REVIEW COMMITTEE, OR REVIEWERS' WRITTEN CRITIQUES, ON THE FOLLOWING ISSUES:

PROTECTION OF HUMAN SUBJECTS (Resume): ACCEPTABLE

INCLUSION OF WOMEN PLAN (Resume): ACCEPTABLE

INCLUSION OF MINORITIES PLAN (Resume): ACCEPTABLE

INCLUSION OF AHRQ PRIORITY POPULATIONS PLAN (Resume): ACCEPTABLE

COMMITTEE BUDGET RECOMMENDATIONS: The budget was recommended as requested. However, the following budget modifications were recommended: 1) It is unclear who will perform the bulk of the data collection in Aims 1, 2, and 3; and 2) Travel budget seems high. The applicant should provide an adequate justification for the proposed budget.
